# A semi-automatic cell type annotation method for single-cell RNA sequencing dataset

**DOI:** 10.5808/GI.2020.18.3.e26

**Published:** 2020-09-08

**Authors:** Wan Kim, Sung Min Yoon, Sangsoo Kim

**Affiliations:** Department of Bioinformatics and Life Science, Soongsil University, Seoul 06978, Korea

**Keywords:** cell type annotation, co-expression network, regulatory network, single-cell RNA sequencing, transcription factor

## Abstract

Single-cell RNA sequencing (scRNA-seq) has been widely applied to provide insights into the cell-by-cell expression difference in a given bulk sample. Accordingly, numerous analysis methods have been developed. As it involves simultaneous analyses of many cell and genes, efficiency of the methods is crucial. The conventional cell type annotation method is laborious and subjective. Here we propose a semi-automatic method that calculates a normalized score for each cell type based on user-supplied cell type–specific marker gene list. The method was applied to a publicly available scRNA-seq data of mouse cardiac non-myocyte cell pool. Annotating the 35 t-stochastic neighbor embedding clusters into 12 cell types was straightforward, and its accuracy was evaluated by constructing co-expression network for each cell type. Gene Ontology analysis was congruent with the annotated cell type and the corollary regulatory network analysis showed upstream transcription factors that have well supported literature evidences. The source code is available as an R script upon request.

## Introduction

Next-generation sequencing technology has transformed transcriptomics by allowing simultaneous identification and quantification of the expressed RNA molecules. However, this RNA-sequencing applied to bulk samples provides averaged expression profiles, not single-cell level variations. On the other hand, single-cell RNA-seq (scRNA-seq) isolates single cells from a given bulk sample, and measures the expression profile of a number of RNA species from each cell, offering the potential of cell-type characteristics as well as cell-type profiles of the bulk sample [1-3]. scRNA-seq has been applied to various fields such as neurobiology and cancer biology [1]. For example, immunotherapy has been developed as an effective cancer therapy, and thus underpinning of cancer immunology is crucial in the development of immunotherapy; scRNA-seq is a useful tool in this area [4-6].

scRNA-seq involves simultaneous analyses of many cell and genes, efficiency is crucial. One of the time-consuming steps is cell type annotation. The conventional method is laborious and subjective. To address this problem, some annotation methods have been developed by using additional transcriptomic data as reference coupled with machine-learning technique [7,8]. For instance, SingleR, a cell type annotation tool for scRNA-seq, leverages reference transcriptomic datasets of pure cell types to infer the cell of origin of each of the single cells independently [7]. But these methods require additional data and consume much computing power needed to perform machine-learning. Here we propose a semi-automatic method that calculates a normalized score for each cell type based on user-supplied cell type–specific marker gene list. The user can easily decide a cut-off score for each cell type based on the plots of score distributions.

## Methods

### Materials

We downloaded raw scRNA-seq datasets from the publicly available ArrayExpress DB (E-MTAB-6173) and Gene Expression Omnibus (GSE92332). A 10× Genomics [3] technology was used to generate these two datasets. One dataset (E-MTAB-6173) was generated from male and female mouse cardiac cell pools after depleting endothelial cells to 10% [9]. The other dataset (GSE92332) was measured from small intestinal epithelial cells from female and male mice that were randomly assigned to treatment groups after matching for the sex and age of 7–10 weeks [10].

### Overall analysis workflow

**Fig. 1.**
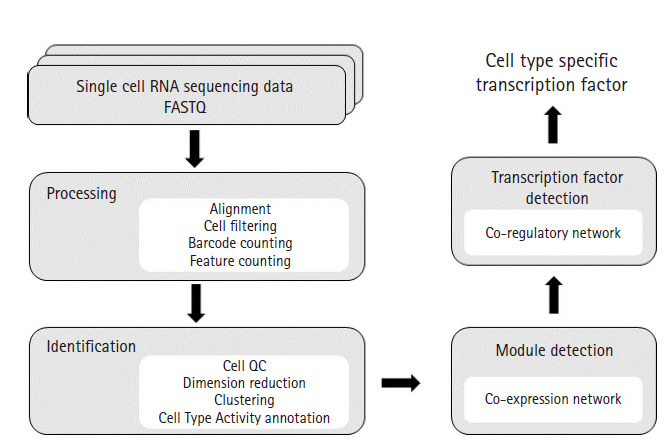
Analysis workflow diagram. This diagram illustrates overall workflow used in our study. FASTQ files were processed for alignment, cell filtering, UMI count and feature (genes) count by using CellRanger. CellRanger is a popular pipeline that processes Chromium single-cell 3′ RNA-sequencing data. Next, Seurat was used to generate clusters of cells. Seurat is an R package offering functions for secondary analysis such as cell QC, dimension reduction, clustering and differential expressed gene analysis. After clustering, we used our Cell Type Activity estimation technique for cell type annotation. To identify modules from each cell type, WGCNA was used. WGCNA is an R package that calculates correlation weighted network by using gene expression data and creates clusters of genes. Enrichment analysis study was conducted on each modules and iRegulon was used to identify potential transcription factors co-regulating the gene sets.

Our scRNA-seq analysis pipeline is based on a well-established practice of processing 10× Genomics data. The sequencing data were processed with Cellranger to obtain an expression matrix of RNAs for each cell. Subsequent processing was performed with Seurat for various quality control steps involving cell filtering, normalization, and removal of technical variation, followed by preliminary analyses such as dimension reduction, clustering, cell type annotation, and differential expression analysis [11]. Our Cell Type Activity (CTA) annotation method is an alternative to the cell type annotation step in Seurat. For each cell type, the expression matrix of gene-by-cell was used in co-expression network analysis using WGCNA [12]. The modules in the network were analyzed for the enrichment of Gene Ontology terms and identification of upstream transcription factors (TFs) using iRegulon (Fig. 1) [13].

### Alignment and pre-filtering

STAR was used to map FASTQ reads to mm10-3.0.0 mouse reference genome [14]. Cellranger detects the cases where two cells are captured by a 10× Genomics GEM bead and filters the RNA counts originated from the dead cells. Using default options of Cellranger 3.0.2, the feature-barcode matrix was generated.

### Dimension reduction, clustering, and annotation

Seurat 3.1.0 was used for principal component analysis, t-stochastic neighbor embedding (t-SNE), clustering, and cell-type annotation of the feature-barcode matrix that had been generated by Cellranger. With the clustered result, each cluster is annotated with a cell type by identifying the dominant expression of a list of cell type–specific markers. The conventional method requires manual examination of a violin plot for each marker of a cell type, followed by confirmation of its high expression in the feature plot. This is a laborious step, often producing imprecise results especially for the cases having a high number of clusters or many similar cell types. In order to reduce human errors, we automated the cell type annotation step using the algorithm that has been well developed for gene-set analysis [15]. Our method is called Cell Type Activity (CTA) method.

The inputs to CTA comprise a feature-barcode matrix, the cluster membership of each cell, and a list of markers for each cell type of the user’s choice. Suppose the following: there are *K* clusters from the Seurat result and these clusters would be annotated with *C* different cell types. If each cell type has N marker genes, there would be a total of *C × N* markers. The following steps are repeated *C* times, one for each cell type. For a given cell type, the first step tabulates the cluster median expression of each marker, generating an *N × K* matrix. Each row of the matrix (*P*) is then normalized so that it sums to unity ($\sum_{k=1}^{K}p_{ik}=1,\;i=1,\cdots ,N$). In the second step, we calculate the weight to emphasize informativeness of a marker gene in classifying the clusters. To calculate this, we use the concept of Gini impurity that measures how homogeneous the groupings are. Because the original Gini impurity reaches its minimum (zero) when only one class is classified, we modified it to give the highest score if a marker gene is expressed specifically in one cluster. The weight for the *i*^th^ gene is defined as below:

(1) $$W_{i}=1+\sum_{k=1}^{K}{p_{ik}}^{2}$$

where *p*_ik_ represents one of the elements of the aforementioned *P* matrix. In the third step, a CTA score for the *k*_th_ cluster, *S_k_*, is calculated as follows:

(2) $$S_{k}=\frac{\sum_{i=1}^{N}E_{ik}\cdot W_{i}}{\sqrt[{3}]{N}}$$

where ${\bar{E}}_{ik}$ is the average expression value of the *i*^th^ marker gene in the *k*^th^ cluster and *W*_i_ is the weight calculated above (Eq. 1). Lastly, we convert the CTA scores to probabilities by normalizing them to a sum of unity. The user decides a cut-off score based on a cumulative normal distribution curve of the normalized CTA scores. The clusters having the score above the cut-off are then annotated with the given cell type. The whole process is repeated for each cell type.

### Co-expression and co-regulatory networks

WGCNA 1.68 was used to construct cell type–specific co-expression networks. The Seurat output was parsed to be used as an input to WGCNA. The resulting modules were further analyzed for the enrichment of Gene Ontology terms.

For the co-expressed modules, the potential upstream TFs were inferred using iRegulon 1.3 available as an application of Cytoscape. For the TF binding motif search, the 20 kb upstream region of transcription start site of each gene was used.

## Results

**Fig. 2.**
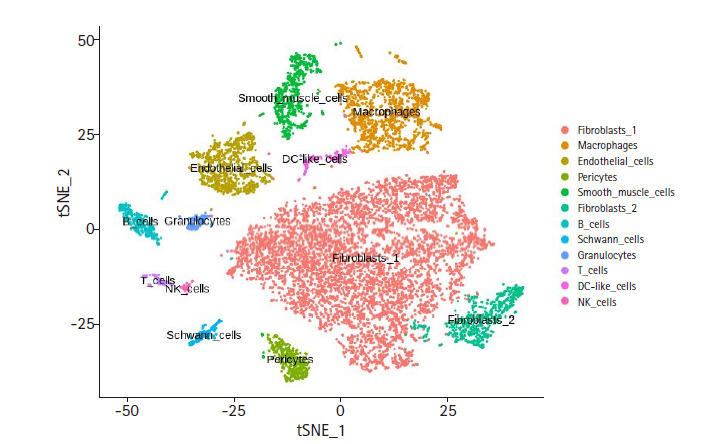
t-distributed stochastic neighbor embedding (t-SNE) result of cardiac non-myocytes. This plot displays final cell type annotation based on Cell Type Activity (CTA) scores. Feature-barcode matrix from single-cell RNA sequencing data was used for dimension reduction by using principal component analysis. After dimension reduction, t-SNE was used to visualize the clusters. The cell type for each cluster was inferred from the CTA score.

The FASTQ files of mouse cardiac cell pools (ArrayExpress E-MTAB-6173) were processed with Cellranger, resulting in the feature-barcode matrix of 11,701 cells. Seurat was used to analyze the single-cell level heterogeneity. The cells having mitochondrial RNAs more than 25% of the total expressed RNAs were removed. In order to remove cells having unrealistic RNA varieties, only the cells with unique feature counts within the range between 200 and 5,000 were kept, resulting in a total of 11,587 cells and 17,432 genes. For the t-SNE clustering, 24 dimensional components as inferred from the principal component analysis and the resolution value of 2.0 were used, resulting in a total of 35 clusters. In order to annotate an appropriate cell type to each cluster we employed the CTA method (see Methods) using 12 cell types having 10 unique marker genes per cell type (Supplementary Table 1) [9]. The CTA score distribution for each cell type was manually examined to adjust the cut-off (see Supplementary Fig. 1A–F for exemplary plots). The t-SNE clustering with the cell type annotation is shown in Fig. 2, while the number of cells and genes identified for each cell type are listed in Table 1. The CTA matrix (12 rows by 35 columns) is given in Supplementary Table 2.

The performance of our CTA annotation method was evaluated with the other scRNA-seq dataset of mouse small intestinal epithelium [10]. The same workflow was applied for cell QC, resulting in a total of 5,188 cells and 14,259 genes. For clustering and visualization, 12 principal components and the resolution value of 0.8 were used, resulting in a total of 15 clusters. We performed the CTA method by using the 11 cell-type lists (Supplementary Table 3). Cumulative distribution of the CTA score was visualized for decision of the cut-off (Supplementary Fig. 2A–K). The final annotation graph visualized by t-SNE is shown in Supplementary Fig. 3, and the CTA matrix is given in Supplementary Table 4. The annotation results for the major clusters were qualitatively congruent with the original work [10].

In order to assess the quality of our CTA annotation method, we performed co-expression and co-regulatory network analyses with the clustering and annotation results of the mouse cardiac cell pools [9]. While the member cells in each cell-type cluster display a similar expression profile as they are clustered together, their expression profiles are not identical to one another, and in fact they show some discrepancy as shown by the volume encompassed by each cluster (Fig. 2). This variation within a cell-type cluster may be an ideal resource for inferring co-regulatory networks. If our CTA method performed well, the resulting network should have reasonable literature evidence congruent with the annotated cell type. For each of the 12 cell-type clusters, co-expression network was constructed with WGCNA. The network was modularized using the soft threshold value (Supplementary Table 5) that produced a scale-free network for each cell-type cluster. Each co-expression module was further analyzed for the enrichment of Gene Ontology terms (Table 2).

**Fig. 3.**
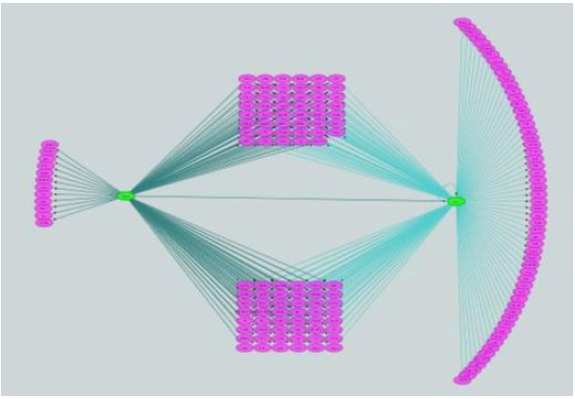
Dendritic cell exemplary module regulatory network. Co-expression network was conducted to find modules of dendritic cell. Gene list in the module that highly enriched immune functions was analyzed to identify co-regulating transcription factors. Network visualization illustrates interactions between transcription factors (TFs; green) and genes (pink) from the module. The analysis revealed that two TF (*CEBPB* and *STAT1*) are significantly related to the genes. There is also relationship between two TFs and self-regulatory loop for the downstream *STAT1*.

As shown in Table 3, an exemplary module from the dendritic cell (DC) cluster was significantly enriched with immune-related terms that are related to the features of dendritic cells such as immune cell activation, recognition of pathogen (the full listing in Supplementary Table 6). This module comprises of 242 genes and the iRegulon motif analysis of their 20 kb upstream of transcription start site inferred 11 potential TFs (Table 4). Among the inferred TFs, *STAT1* and *CEBPB* have more than 100 target genes each. Their regulatory relationship is depicted in Fig. 3, showing that *CEBPB* regulates *STAT1*, which is also self-regulated. Having established this, their roles in DC biology was surveyed from the literature. It is known that up-regulation of *STAT6* pathway plays an important role in the differentiation of immature DCs, and its down-regulation is related to the maturation of DCs. It is reported that *STAT1* pathway works opposite to *STAT6* pathway, maturating mDCs [16]. *IRF8*, an epigenetic and fate-determining TF of plasmacytoid dendritic cell (pDC), modulates chromatin modification of thousands of pDC enhancers. *CEBPB* forms a negative feedback loop with *IRF8*, determining the epigenetic fate of monocyte-derived DCs [17].

## Discussion

Here we propose an efficient semi-automatic processing pipeline of scRNA-seq data, called CTA method. Its quality was assessed by constructing co-expression and co-regulatory networks using the cell type annotation results. Our results were qualitatively congruent with the literature information. In scRNA-seq, many really expressed RNA species are missed. If this drop-out event can be complemented by imputation, much richer information can be retrieved. However, the current implementation of the imputation such as BISCUIT is very slow and not attempted in this work [18].

The strategy demonstrated in this work may find useful applications in inferring regulators of various cell types. For example, for the cell types whose critical differentiation regulators are elusive, co-regulatory network construction of progenitor and differentiated cells may elucidate key modules for the differentiation.

## Figures and Tables

**Table 1.** Annotation summary table

	Cell	Gene
Fibroblast 1	6386	16840
Macrophage	1427	15773
Endothelial cell	920	14594
Smooth muscle cell	712	14616
Fibroblast 2	661	15324
Pericyte	425	12968
B cell	331	12646
Dendritic cell	208	13007
Schwann cell	179	12416
Granulocyte	134	9392
T cell	121	11282
Natural killer cell	56	10187

This chart displays summary of cell types after annotation based on Cell Type Activity score. The amount of cells of each cell type is written at cell column. The number of genes that were expressed in the cell type is showed at gene column.

**Table 2.** Co-expression module summary table

Cell type	All modules	GO assigned modules
Dendritic cell	98	13
T cell	86	5
Schwann cell	81	7
Pericyte	81	2
Granulocyte	76	6
Endothelial cell	67	13
Smooth muscle cell	57	11
B cell	50	7
Natural killer cell	46	7
Macrophage	37	14
Fibroblast 2	23	5
Fibroblast 1	12	8

Each column represents the number of modules generated by co-expression network and the number of modules that were enriched Gene Ontology (GO) term, respectively.

**Table 3.** Dendritic cell exemplary module GO term result

Enrichment P	Term ID	Term name
5.94E-24	GO:0035456	Response to interferon-beta
2.15E-20	GO:0006952	Defense response
3.99E-20	GO:0045087	Innate immune response
1.61E-19	GO:0035458	Cellular response to interferon-beta
4.47E-19	GO:0043207	Response to external biotic stimulus
4.47E-19	GO:0051707	Response to other organism
1.96E-15	GO:0098542	Defense response to other organism

Enrichment analysis revealed the module that is highly associated with immune Gene Ontology (GO) term. This table represents the list of GO terms from tan module.

**Table 4.** Dendritic cell inferred TF summary table

Inferred TF	Target gene count
Stat1	156
Cebpb	109
Ar	19
Sox9	32
Atf2	21
Yy1	14
Cebpe	8
Mybl2	10
Snai2	9
Tbx15	27
Bdp1	12

The gene set from the exemplary module of dendritic cell was used for regulatory network analysis, which enables to infer co regulation transcription factor (TF). This chart shows revealed TF names and related gene from the module.

## Supplementary Materials

Supplementary Fig. 1. These plots show cumulative normal distribution of Cell Type Activity (CTA) scores for cardiac non-myocytes. Red lines show threshold for determining cell type of clusters. Dots indicate the clusters (A–F).

Supplementary Fig. 2. These plots show cumulative normal distribution of Cell Type Activity (CTA) scores for small intestine epithelial cells. Red lines show threshold for determining cell type of clusters. Dots indicate the clusters (A–K).

Supplementary Fig. 3. This graph displays final cell type annotation based on Cell Type Activity score of small intestine epithelial cell clusters. Principal component analysis was used for dimension reduction and t-stochastic neighbor embedding (t-SNE) is used for visualization.

Supplementary Table 1. Unique marker genes of 12 cell types for mouse cardiac cells

Supplementary Table 2. Cell Type Activity score matrix of male and female mouse cardiac cell clusters

Supplementary Table 3. Unique marker genes of 11 cell types for small intestinal epithelial cells

Supplementary Table 4. Cell Type Activity score matrix of male and female mouse small intestinal epithelial cell clusters.

Supplementary Table 5. Soft threshold values for network modularization

Supplementary Table 6. Gene Ontology term full text of a dendritic cell exemplary module
