# Supplementary material for: A semi-automatic cell type annotation method for single-cell RNA sequencing dataset
Source: Genomics Inform. 2020 Sep 8;18(3):e26. doi: 10.5808/GI.2020.18.3.e26 (PMC7560448; doi:10.5808/GI.2020.18.3.e26)
Supplement: Supplementary Fig. 1. — These plots show cumulative normal distribution of Cell Type Activity (CTA) scores for cardiac non-myocytes. Red lines show threshold for determining cell type of clusters. Dots indicate the clusters (A–F). [file gi-2020-18-3-e26-suppl1.pdf]

A

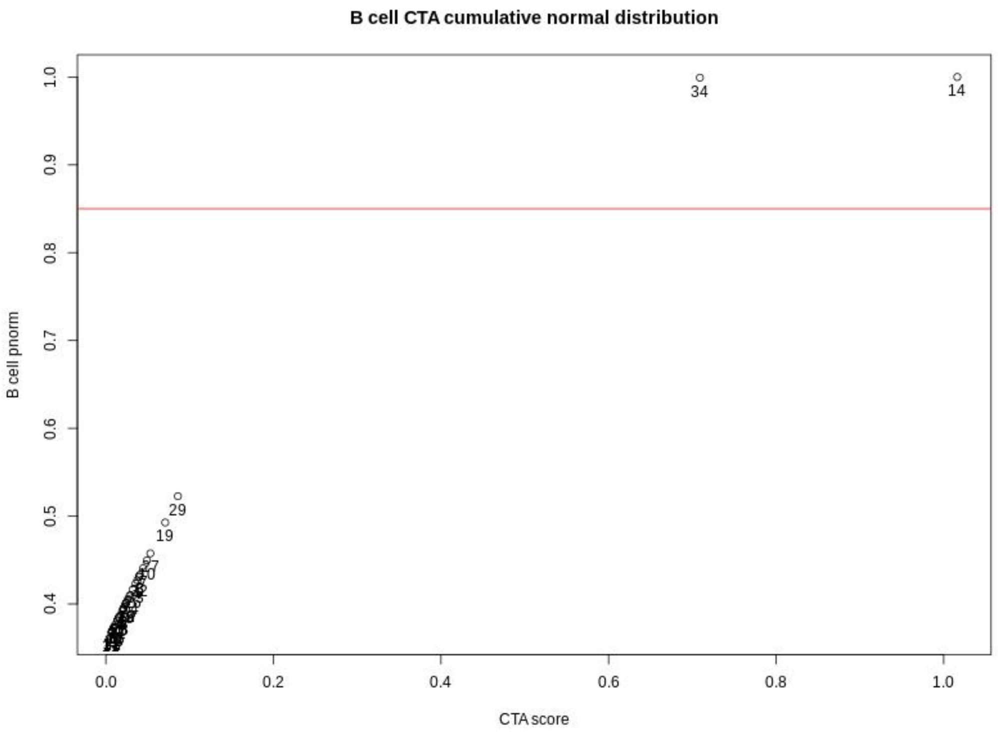
B cell CTA cumulative normal distribution
B cell pnorm
CTA score
34
14
29
19
1.0
0.9
0.8
0.7
0.6
0.5
0.4
0.0
0.2
0.4
0.6
0.8
1.0

B

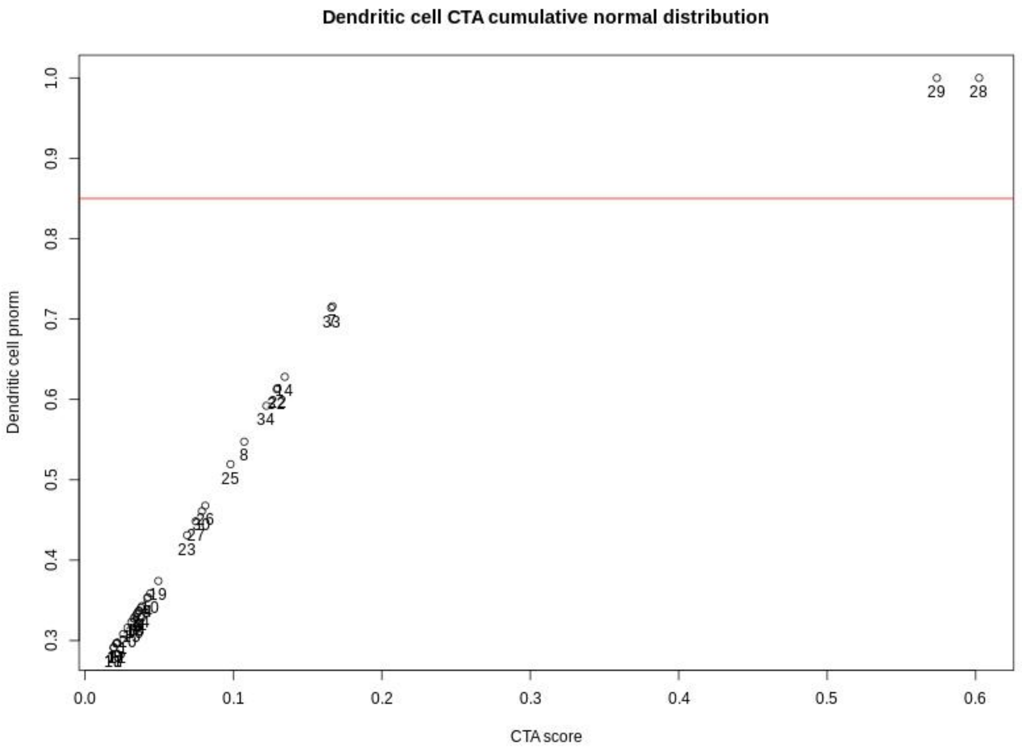
Dendritic cell CTA cumulative normal distribution
Dendritic cell pnorm
CTA score
29
28
33
34
8
25
27
23
19
1.0
0.9
0.8
0.7
0.6
0.5
0.4
0.3
0.0
0.1
0.2
0.3
0.4
0.5
0.6

C

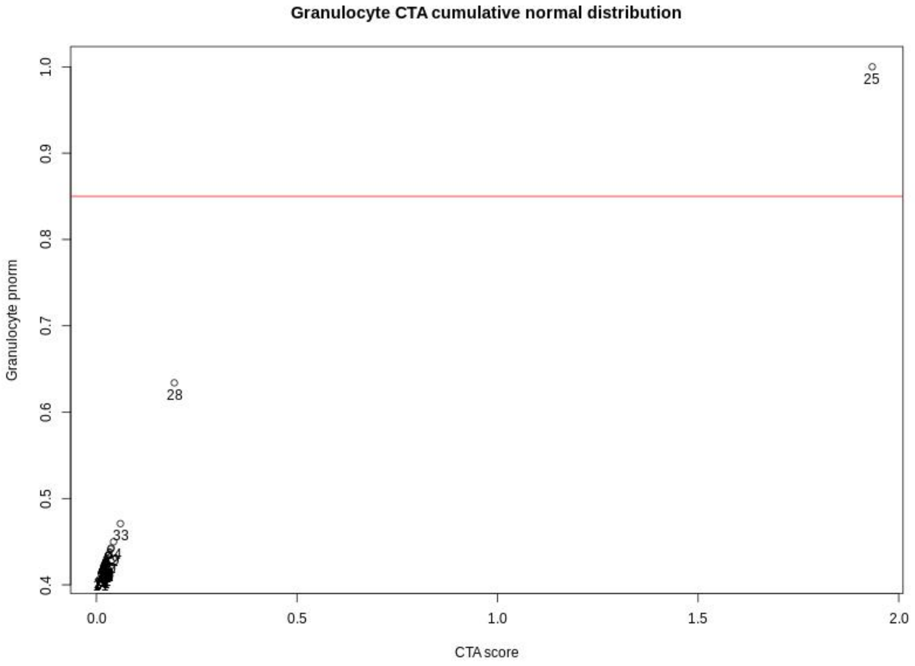

D

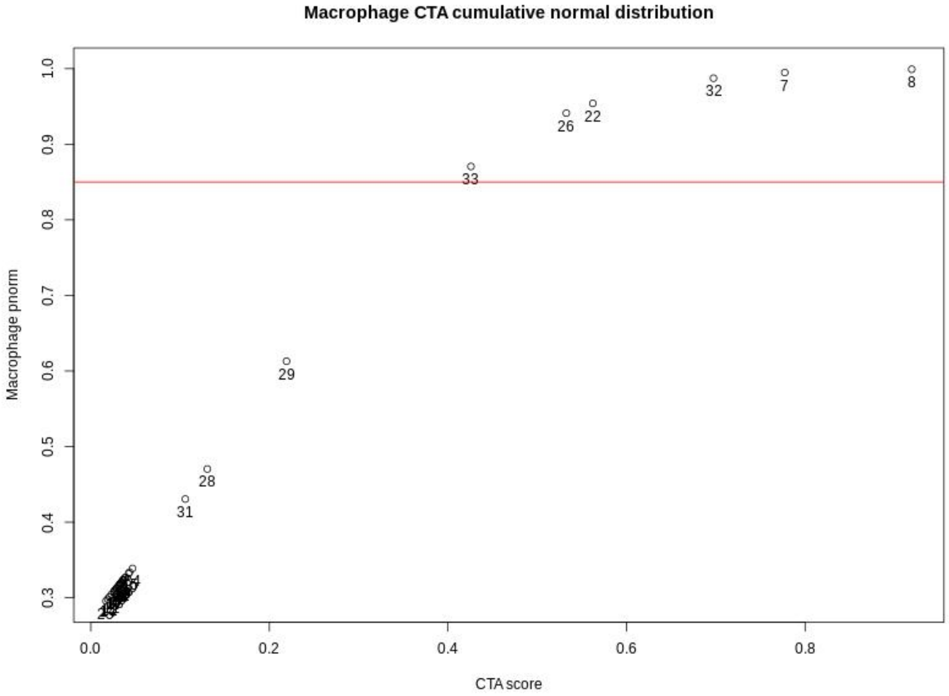

Supplemenatry **E**

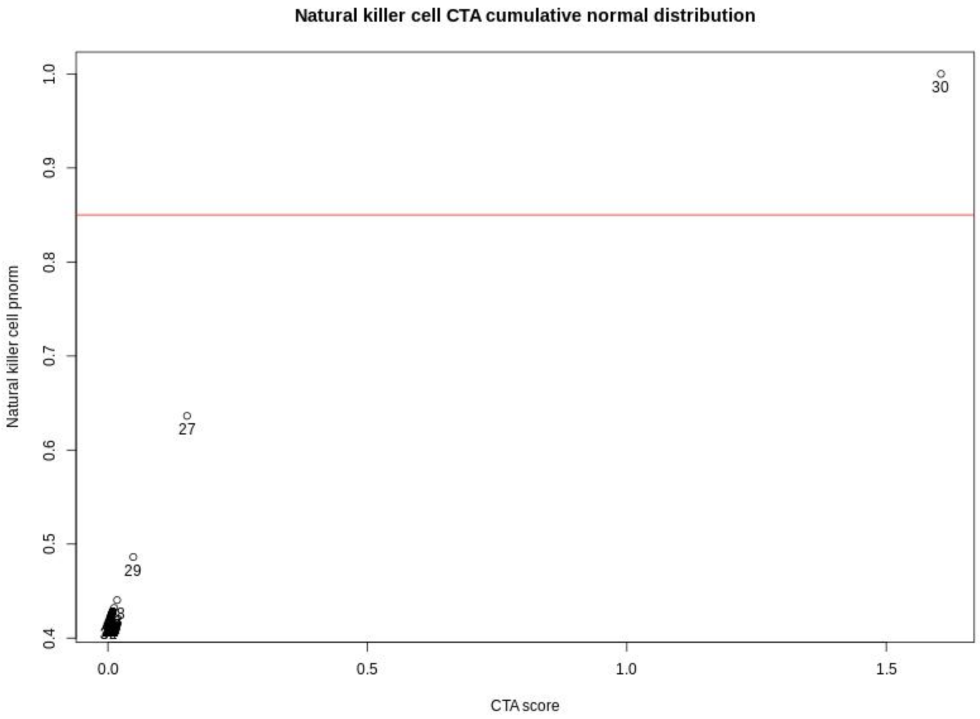

F

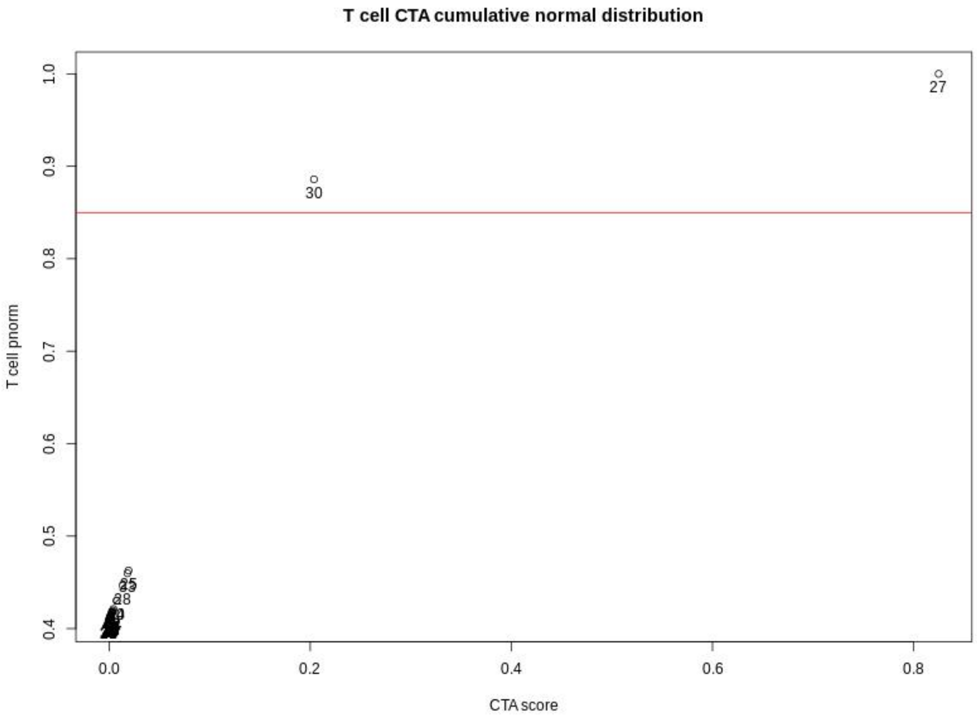

Supplementary Fig. 1. These plots show cumulative normal distribution of Cell Type Activity (CTA) scores for cardiac non-myocytes. Red lines show threshold for determining cell type of clusters. Dots indicate the clusters (A–F).
