# Supplementary material for: A semi-automatic cell type annotation method for single-cell RNA sequencing dataset
Source: Genomics Inform. 2020 Sep 8;18(3):e26. doi: 10.5808/GI.2020.18.3.e26 (PMC7560448; doi:10.5808/GI.2020.18.3.e26)
Supplement: Supplementary Fig. 2. — These plots show cumulative normal distribution of Cell Type Activity (CTA) scores for small intestine epithelial cells. Red lines show threshold for determining cell type of clusters. Dots indicate the clusters (A–K). [file gi-2020-18-3-e26-suppl2.pdf]

A

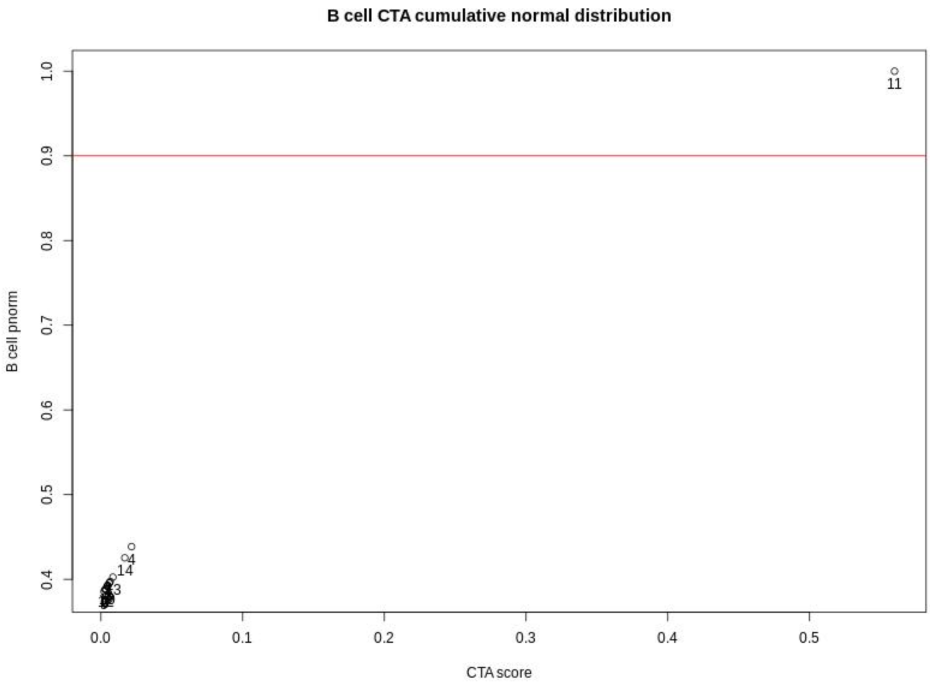
B cell CTA cumulative normal distribution
B cell pnorm
CTA score
11
14
4
13
0.0
0.1
0.2
0.3
0.4
0.5
0.4
0.5
0.6
0.7
0.8
0.9
1.0

B

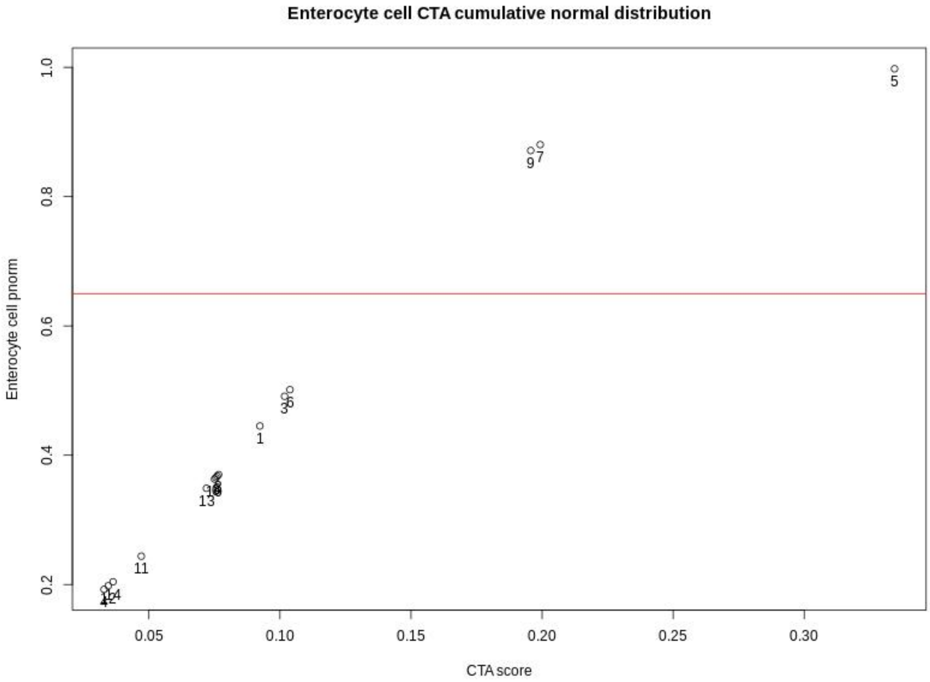
Enterocyte cell CTA cumulative normal distribution
Enterocyte cell pnorm
CTA score
5
9
7
3
6
1
13
11
0.05
0.10
0.15
0.20
0.25
0.30
0.2
0.4
0.6
0.8
1.0

C

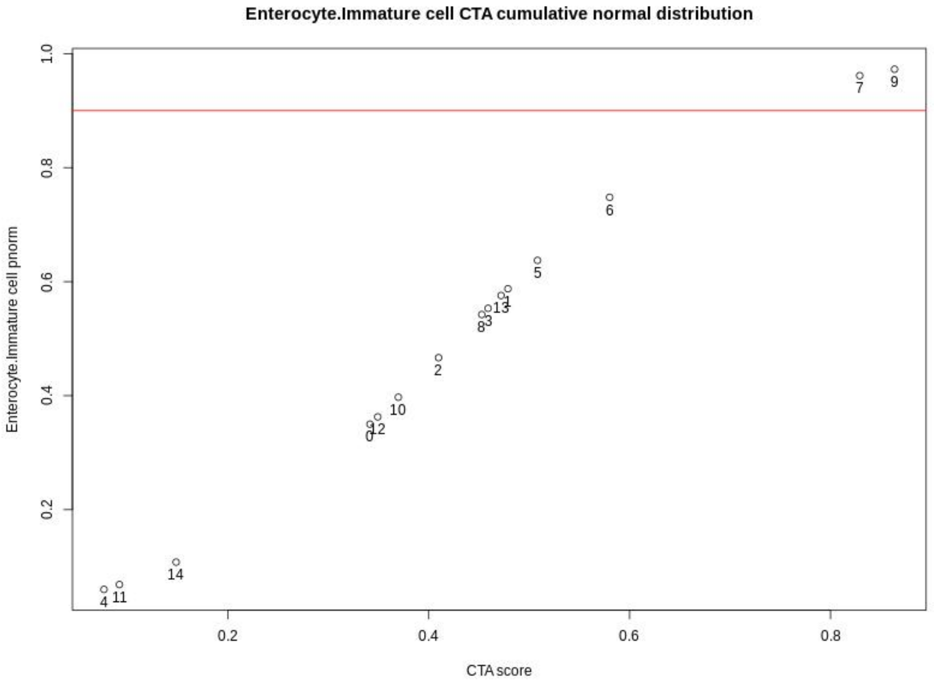

Enterocyte.Immature cell CTA cumulative normal distribution
Enterocyte.Immature cell pnorm
CTA score
0.2
0.4
0.6
0.8
1.0

D

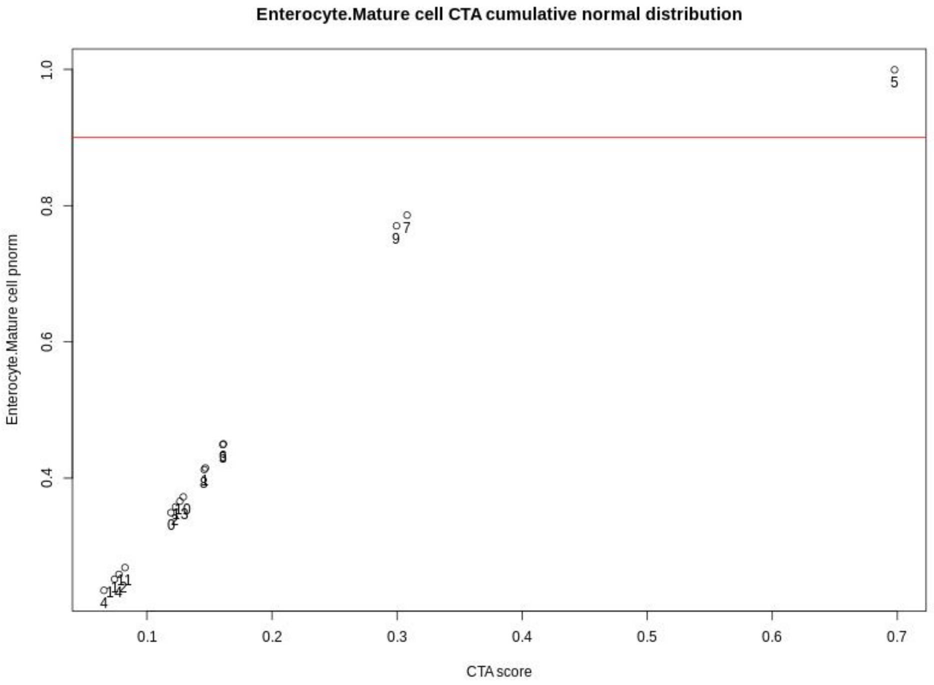

Enterocyte.Mature cell CTA cumulative normal distribution
Enterocyte.Mature cell pnorm
CTA score
0.1
0.2
0.3
0.4
0.5
0.6
0.7
0.8
1.0

E

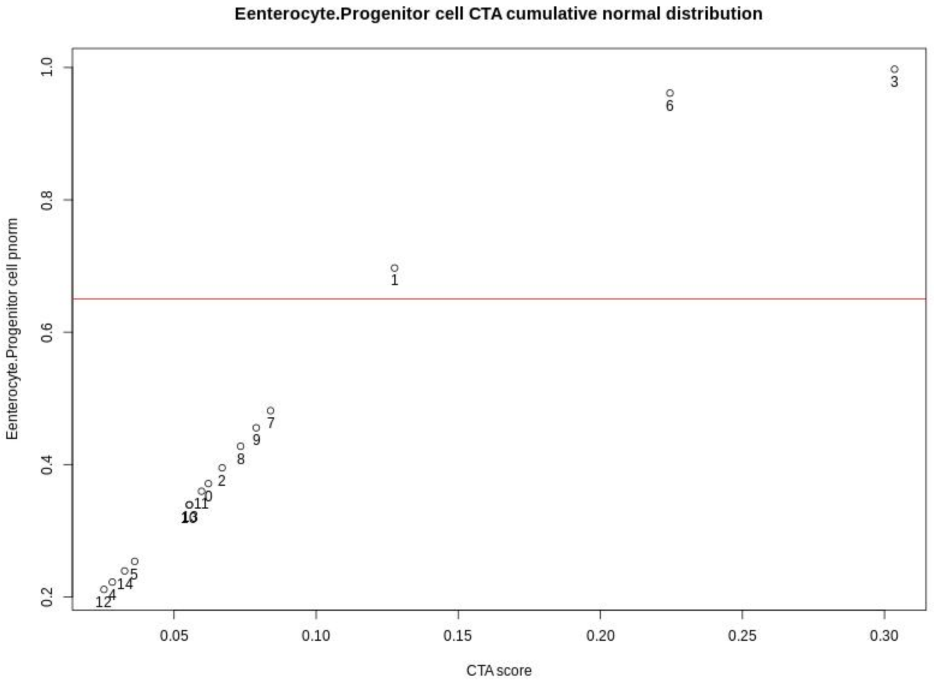
Eenterocyte.Progenitor cell CTA cumulative normal distribution
Eenterocyte.Progenitor cell pnorm
CTA score

F

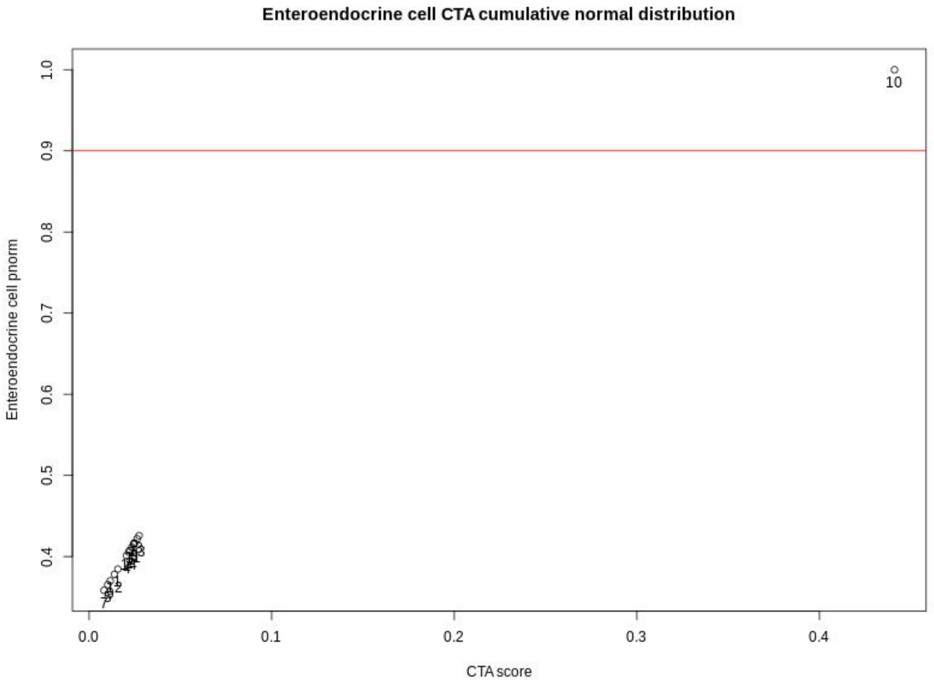
Enteroendocrine cell CTA cumulative normal distribution
Enteroendocrine cell pnorm
CTA score

G

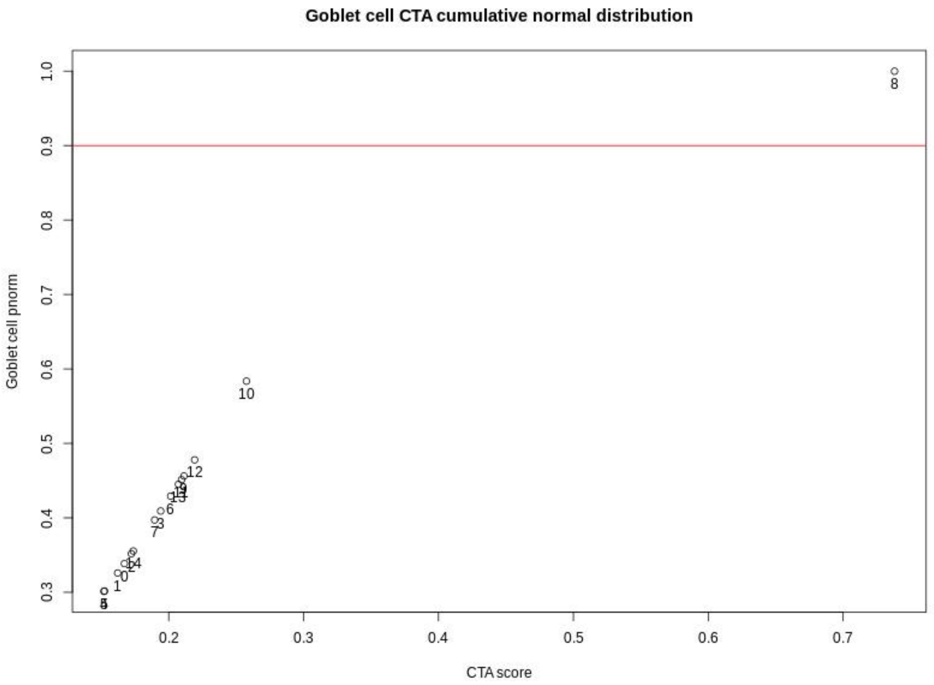
Goblet cell CTA cumulative normal distribution
Goblet cell pnorm
CTA score
8
10
12
6
3
7
1
0
14

H

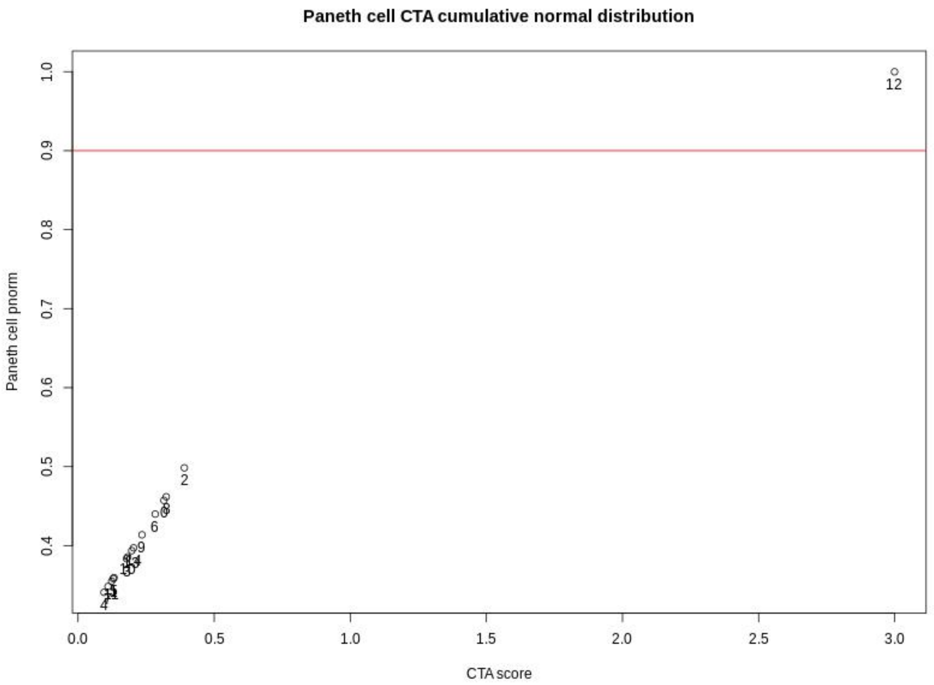
Paneth cell CTA cumulative normal distribution
Paneth cell pnorm
CTA score
12
2
6
9
4

I

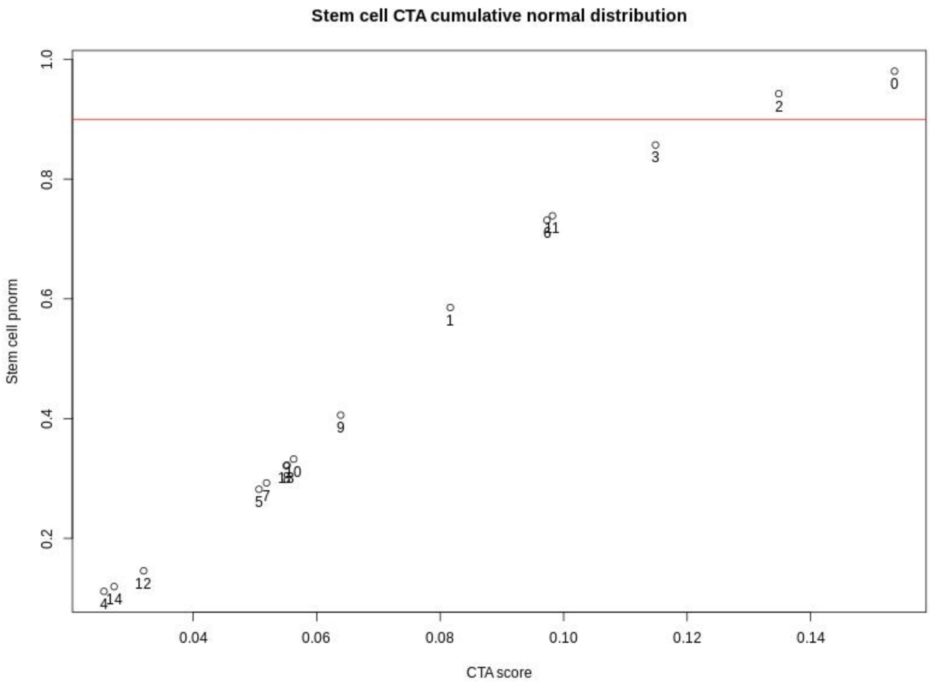
Stem cell CTA cumulative normal distribution
Stem cell pnorm
CTA score
0.2
0.4
0.6
0.8
1.0
0.04
0.06
0.08
0.10
0.12
0.14
0
2
3
11
6
1
9
10
5
7
12
4
14

J

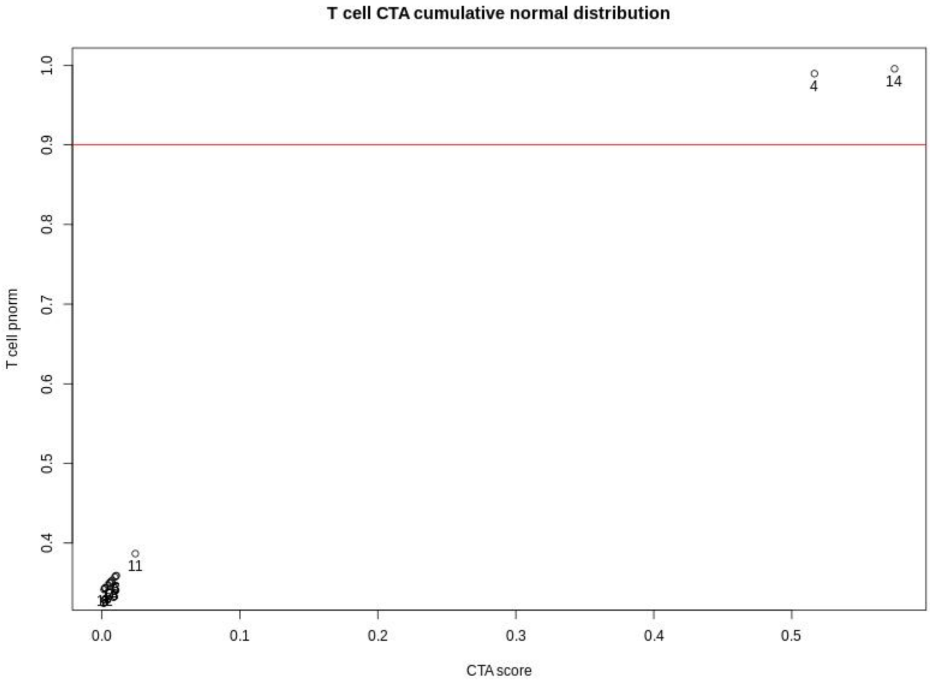
T cell CTA cumulative normal distribution
T cell pnorm
CTA score
0.4
0.5
0.6
0.7
0.8
0.9
1.0
0.0
0.1
0.2
0.3
0.4
0.5
4
14
11

K

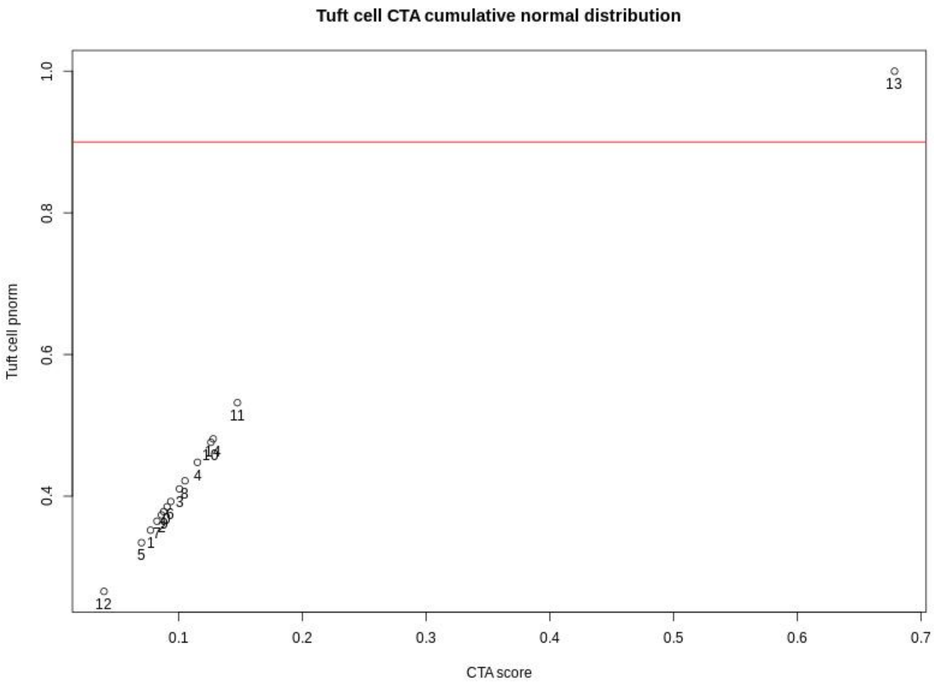

Supplementary Fig. 2. These plots show cumulative normal distribution of Cell Type Activity (CTA) scores for small intestine epithelial cells. Red lines show threshold for determining cell type of clusters. Dots indicate the clusters (A–K).
