# Supplementary material for: A semi-automatic cell type annotation method for single-cell RNA sequencing dataset
Source: Genomics Inform. 2020 Sep 8;18(3):e26. doi: 10.5808/GI.2020.18.3.e26 (PMC7560448; doi:10.5808/GI.2020.18.3.e26)
Supplement: Supplementary Fig. 3. — This graph displays final cell type annotation based on Cell Type Activity score of small intestine epithelial cell clusters. Principal component analysis was used for dimension reduction and t-stochastic neighbor embedding (t-SNE) is used for visualization. [file gi-2020-18-3-e26-suppl3.pdf]

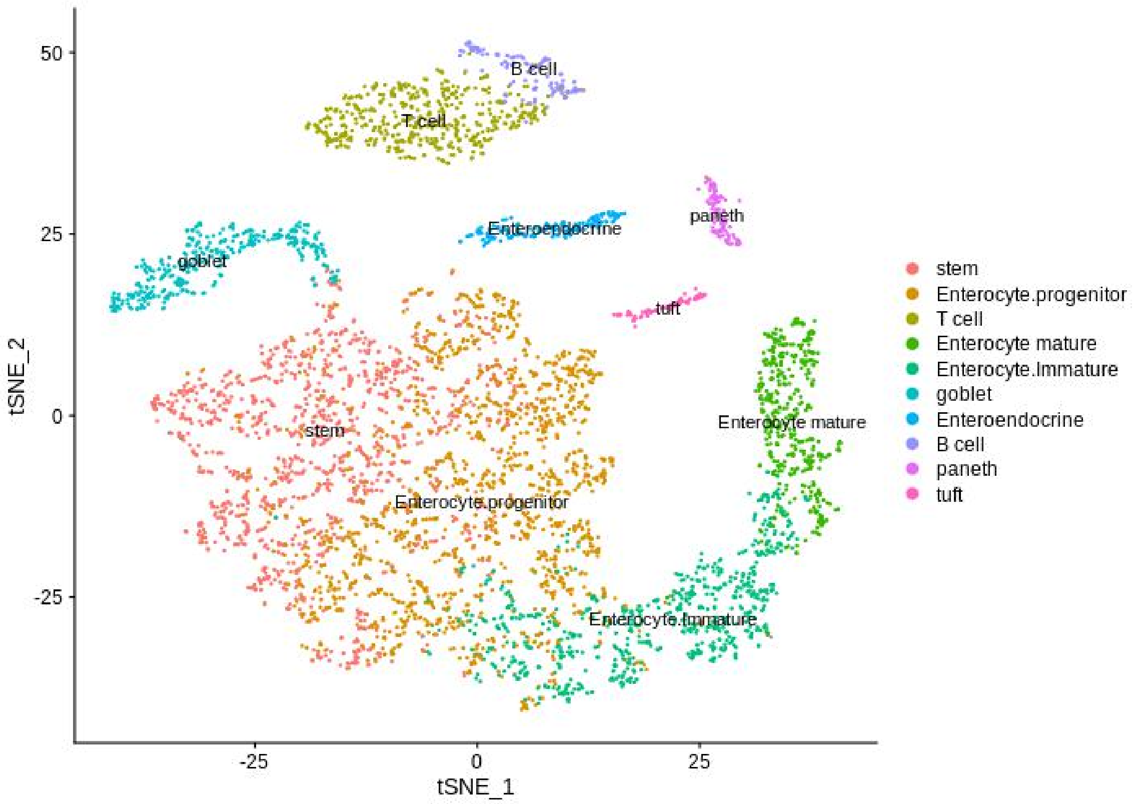

Supplementary Fig. 3. This graph displays final cell type annotation based on Cell Type Activity score of small intestine epithelial cell clusters. Principal component analysis was used for dimension reduction and t-stochastic neighbor embedding (t-SNE) is used for visualization.
