# Supplementary material for: A semi-automatic cell type annotation method for single-cell RNA sequencing dataset
Source: Genomics Inform. 2020 Sep 8;18(3):e26. doi: 10.5808/GI.2020.18.3.e26 (PMC7560448; doi:10.5808/GI.2020.18.3.e26)
Supplement: Supplementary Table 1. — Unique marker genes of 12 cell types for mouse cardiac cells [file gi-2020-18-3-e26-suppl4.pdf]

Supplementary Table 1. Unique marker genes of 12 cell types for mouse cardiac cells

B cell	Dendritic cell	Endotherial cell	Fibroblast 1	Fibroblast 2	Granulocyte	Macrophage	Nutral killer cell	Perycyte	Schwann cell	Smooth muscle cell	T cell
Cd79a	Lgals3	Ly6c1	Lamc1	Dkk3	S100a8	Dab2	Ccl5	Kcnj8	Plp1	Tagln	Cd3g
Ly6d	Napsa	Egfl7	Pcsk6	Tbx20	S100a9	Adgre1	Nkg7	Vtn	Kcna1	Mustn1	Cd3d
Cd79b	Plbd1	Gpihbp1	Pdgfra	Wif1	Slpi	Mgl2	Klrk1	Colec11	Cnp	Myh11	Lat
H2DMb2	Ccr2	Cdh5	Entpd2	Frzb	Csf3r	Mrc1	Klre1	Steap4	S100b	Mylk	Cd3e
Ms4a1	Rnase6	Mgll	Dpep1	Meox1	Hcar2	Hpgd	Klrd1	Abcc9	Gfra3	Pcp4l1	Skap1
H2Ob	Plac8	Slc9a3r2	Adamts5	Prg4	Lmnb1	P2ry6	Ncr1	Myo1b	Gpr37l1	Sncg	Il7r
Fcmr	Ifitm6	Emcn	Medag	Abi3bp	Retnlg	C3ar1	Ctsw	Cog7	Nrn1	Lmod1	Lef1
Ccr7	Naaa	Kdr	Ms4a4d	Pdgfrl	Clec4d	F13a1	Klrb1c	P2ry14	Aspa	Des	Cd247
Bank1	Ear2	Pecam1	Lamb1	Mdk	Hp	Maf	Gzma	Heyl	Cd59a	Pln	Tcf7
Cd55	H2afy	Rgcc	Tcf21	Gstm5	Hdc	Ms4a7	Gzmb	Gnb4	Stmn1	Nrip2	Itk
