# Supplementary material for: A semi-automatic cell type annotation method for single-cell RNA sequencing dataset
Source: Genomics Inform. 2020 Sep 8;18(3):e26. doi: 10.5808/GI.2020.18.3.e26 (PMC7560448; doi:10.5808/GI.2020.18.3.e26)
Supplement: Supplementary Table 2. — Cell Type Activity score matrix of male and female mouse cardiac cell clusters [file gi-2020-18-3-e26-suppl5.pdf]

Supplementary Table 2. Cell Type Activity score matrix of male and female mouse cardiac cell clusters

	0	1	2	3	4	5	6	7	8	9	10	11	12	13	14	15	16	17	18	19	20	21	22	23	24	25	26	27	28	29	30	31	32	33	34
**B_LIST**	0.009226	0.005939	0.008021	0.007453	0.014391	0.01525	0.008088	0.017074	0.015622	0.003488	0.018812	0.002481	0.002929	0.003791	0.389371	0.004364	0.009259	0.005323	0.002786	0.027169	0.003557	0.005339	0.009225	0.006204	0.010913	0.01019	0.012365	0.020361	0.008868	0.032957	0.005908	0.002805	0.015154	0.013637	0.271679
**DC_LIST**	0.010878	0.008042	0.012002	0.011377	0.013236	0.013081	0.011077	0.05195	0.033453	0.006812	0.013776	0.006083	0.006683	0.010534	0.041958	0.009759	0.01027	0.006623	0.006063	0.01543	0.008956	0.006704	0.040436	0.021463	0.011771	0.03057	0.02526	0.023262	0.187671	0.178814	0.024577	0.011331	0.040291	0.0517	0.038108
**ENDO_LIST**	0.007934	0.005115	0.008431	0.006723	0.010965	0.009933	0.006789	0.00495	0.007674	0.173271	0.012243	0.006347	0.008942	0.008861	0.006733	0.008831	0.006966	0.005281	0.009279	0.017432	0.167106	0.131836	0.00563	0.013391	0.008069	0.007305	0.008129	0.012845	0.010948	0.00543	0.010698	0.011014	0.108635	0.018949	0.147318
**FIBRO_1_LIST**	0.068324	0.058225	0.053825	0.072326	0.050389	0.070014	0.068577	0.005787	0.006034	0.00689	0.068254	0.010751	0.007969	0.021225	0.005993	0.015461	0.06274	0.056519	0.007698	0.071607	0.007652	0.015532	0.006139	0.01056	0.060358	0.005607	0.020487	0.005894	0.007708	0.006561	0.007112	0.025548	0.007709	0.014624	0.009902
**FIBRO_2_LIST**	0.037013	0.021962	0.047276	0.036077	0.071848	0.028572	0.030176	0.005381	0.006551	0.012999	0.033787	0.01216	0.007879	0.126395	0.005606	0.199759	0.04661	0.018175	0.008111	0.022485	0.010564	0.012915	0.006379	0.014492	0.042112	0.006197	0.038047	0.005771	0.005429	0.007566	0.005019	0.031458	0.0085	0.015268	0.011461
**GRANULO_LIST**	0.006724	0.005305	0.006898	0.007227	0.006394	0.006194	0.006599	0.010243	0.010495	0.008014	0.008227	0.004347	0.005683	0.007398	0.008023	0.007165	0.007651	0.004827	0.006904	0.008268	0.007821	0.005258	0.012277	0.007894	0.005171	0.661488	0.007479	0.008048	0.066443	0.012618	0.01017	0.007567	0.0101	0.020488	0.014591
**MACRO_LIST**	0.006402	0.005469	0.007064	0.007009	0.005978	0.005544	0.00627	0.149285	0.176601	0.007362	0.006927	0.0044	0.00398	0.007417	0.003622	0.008338	0.005934	0.004911	0.005249	0.006686	0.008106	0.006547	0.107999	0.006284	0.006951	0.003265	0.102288	0.005051	0.025061	0.042091	0.006891	0.020325	0.133952	0.081776	0.008963
**NK_LIST**	0.003207	0.002528	0.002909	0.004418	0.003895	0.004223	0.002292	0.004528	0.003825	0.002532	0.00257	0.003091	0.003133	0.003854	0.003384	0.00446	0.00364	0.002487	0.002281	0.00449	0.004497	0.003267	0.004634	0.0028	0.00352	0.003536	0.004706	0.074771	0.008696	0.024012	0.786824	0.002238	0.001418	0.005903	0.001431
**PERY_LIST**	0.021133	0.018637	0.019934	0.028201	0.016746	0.014072	0.020206	0.006859	0.007198	0.016296	0.01256	0.337929	0.047716	0.021417	0.005122	0.016033	0.021362	0.013492	0.026423	0.010326	0.025516	0.0141	0.005952	0.010243	0.017327	0.007057	0.014279	0.004259	0.008714	0.012704	0.008182	0.137903	0.016201	0.018826	0.017075
**SCHWAN_LIST**	0.010619	0.006806	0.013264	0.010254	0.013987	0.009818	0.009924	0.012619	0.013537	0.014528	0.012089	0.005536	0.006422	0.025784	0.018693	0.02808	0.010605	0.00854	0.01108	0.011832	0.022145	0.011004	0.011211	0.519316	0.009534	0.006469	0.012143	0.013588	0.005766	0.013796	0.014265	0.012129	0.015355	0.057431	0.021829
**SMC_LIST**	0.006316	0.004833	0.007352	0.005249	0.009061	0.006523	0.006636	0.006927	0.007496	0.014937	0.008396	0.039047	0.2503	0.013401	0.005593	0.016371	0.00639	0.005169	0.292772	0.011805	0.030366	0.015215	0.008018	0.008092	0.005972	0.005962	0.009763	0.004404	0.006321	0.007579	0.004638	0.133479	0.017063	0.005033	0.01352
**T_LIST**	0.001208	0.000571	0.001907	0.001396	0.001563	0.001138	0.001242	0.002668	0.002222	0.001755	0.002249	0.001015	0.001471	0.001711	0.000619	0.001397	0.001224	0.001011	0.000471	0.00094	0.006687	0.003791	0.001516	0.000762	0.002802	0.016933	0	0.723343	0.011841	0	0.178653	0	0.003242	0.016011	0.006643
