# Supplementary material for: A semi-automatic cell type annotation method for single-cell RNA sequencing dataset
Source: Genomics Inform. 2020 Sep 8;18(3):e26. doi: 10.5808/GI.2020.18.3.e26 (PMC7560448; doi:10.5808/GI.2020.18.3.e26)
Supplement: Supplementary Table 3. — Unique marker genes of 11 cell types for small intestinal epithelial cells [file gi-2020-18-3-e26-suppl6.pdf]

Supplementary Table 3. Unique marker genes of 11 cell types for small intestinal epithelial cells

Goblet	Paneth	Tuft	Enteroendocrine	Enterocyte	Stem	TA	Enterocyte progenitor	Enterocyte Immature	Enterocyte Mature	B cell	T cell
Agr2	Gm15284	Alox5ap	Chgb	Mep1b	Lgr5	Stmn1	1190002F15Rik	Reg3g	2200002D01Rik	Cd79a	Cd3g
Fcgbp	AY761184	Lrmp	Gfra3	Anpep	Gkn3	Tubb5	1810065E05Rik	Gsdmc4	Abcb1a	Ly6d	Cd3d
Tff3	Defa17	Hck	Cck	Gsta1	Ascl2		Arhgef39	Prss32	Ace	Cd79b	Lat
Clca1	Gm14851	Avil	Vwa5b2	Apoa1	Olfm4		Aurka	Krt8	Acsl5	H2DMb2	Cd3e
Zg16	Defa22	Rgs13	Neurod1	Gm3776	Rgmb		Birc5	Casp6	Adh6a	Ms4a1	Skap1
Tpsg1	Defa-rs1	Ltc4s	Fev	Igsf9	Igfbp4		Bub1b		Aldh1a1	H2Ob	Il7r
Muc2	Defa3	Trpm5	Aplp1	Il18	2210407C18Rik		Ccna2		Aldob	Fcmr	Lef1
Galnt12	Defa24	Dclk1	Scgn	Ace2	Jun		Ccnb1		Amn	Ccr7	Cd247
Atoh1	Defa26	Spib	Neurog3	Creb3l3	Pdgfa		Ccnb2		Anpep	Bank1	Tcf7
Rep15	Defa21	Fyb	Resp18	Krt20	Soat1		Cdc20		Anxa2	Cd55	Itk
S100a6	Lyz1	Ptpn6	Trp53i11	Slc9a3	Tnfrsf19		Cdc25c		Aoc1		
Pdia5	Gm15292	Matk	Bex2	Dpep1	Cyp2e1		Cdca3		Apoa4		
Klk1	Mptx2	Snrnp25	Rph3al	Slc25a45	Fstl1		Cdkn2d		Apoc2		
Pla2g10	Ang4	Sh2d7	Scg5	Rbp2	H2-Eb1		Cdkn3		Apoc3		
Spdef		Ly6g6f	Pcsk1	Ms4a18	Ifitm3		Cenpa		Arf6		
Lrrc26		Kctd12	Isl1	Reg3b	Prelp		Cenpe		Aspa		
Ccl9		1810046K07Rik	Maged1	Reg3a	Scn2b		Ckap2l		Cbr1		
Bace2		Hpgds	Fabp5	Clec2h	A930009A15Rik		Ckap5		Ceacam20		
Bcas1		Tuba1a	Celf3	Slc51b	H2-Ab1		Cmc2		Cgref1		
Slc12a8		Pik3r5	Pcsk1n	Cyp2d26	Slc1a2		Cnih4		Ckb		
Smim14		Vav1	Fam183b	Adh6a	Cd74		Fbxl8		Ckmt1		
Tspan13		Tspan6	Prnp	Bco2	Sp5		Gpsm2		Clec2h		
Txndc5		Skap2	Tac1	Slc3a1	Noxa1		Hmmr		Cndp2		
Creb3l4		Pygl	Gpx3	Cyp3a13	Rgcc		Id1		Cox7a1		
C1galt1c1		Ccdc109b	Cplx2	Slc16a5	Sorbs2		Kif22		Creb3l3		
Creb3l1		Ccdc28b	Nkx2-2	Btnl1	Sectm1b		Kif23		Crip1		
Qsox1		Plcg2	Olfm1	2010106E10Rik	H2-Aa		Kif4		Cubn		
Guca2a		Ly6g6d	Vim	Maob	Cdo1		Knstrn		Cyb5a		
Scin		Alox5	Rimbp2	Sis	Slc14a1		Mad2l1		Cyb5b		
Ern2		Pou2f3	Anxa6	Acad11	Clca2		Melk		Cyb5r3		
AW112010		Gng13	Scg3	Edn2	Tifa		Nek2		Cyp2b10		
Fkbp11		Bmx	Ngfrap1	Spink3	Pls3		Pif1		Cyp2c65		
Capn9		Ptpn18	Insm1	H2-Q1	Hmgcs2		Plk1		Cyp2c66		
Stard3nl		Nebl	Gng4	Sult2b1	Arid5b		Rbp7		Cyp2d26		
Slc50a1		Limd2	Pax6	Slc7a7	Agr3		Sapcd2		Cyp3a11		
Sdf2l1		Pea15a	Cnot6l	1700019G17Rik	Slc12a2		Slc16a1		Cyp3a13		
Hgfac		Tmem176a	Cacna2d1	Dgat2	Rassf5		Spc24		Cyp3a25		
Galnt7		Smpx	Tox3	Enpep	Rnf43		Spc25		Dhcr24		
Hpd		Itpr2	Slc39a2	Fmo5	Nrn1		Tacc3		Dhrs1		
Ttc39a		Il13ra1	Riiad1	2010001E11Rik	Lamb3		Tpx2		Dnase1		
Tmed3		Siglecf		Fam3b	Cd44		Ube2c		Dpep1		
Pdia6		Ffar3		Slc26a6	Axin2				Enpep		
Uap1		Rac2		Mpp1	Slc27a2				Ephx2		
Gcnt3		Hmx2		Ces1f	Afap1l1				Fabp1		
Tnfaip8		Bpgm		Apoa4	Ccdc3				Fabp6		
Dnajc10		Inpp5j		Slc5a11	Lrig1				Fam151a		
Ergic1		Ptgs1		2010003K11Rik	Noxo1				Fam213a		
Tsta3		Aldh2		Eci3	Cdk6				Fgf15		
Kdelr3		Pik3cg		Cyp4f14	Amica1				Gda		
Foxa3		Cd24a		Btnl6	Tgif1				Gpd1		
Tpd52		Ethe1		Ace	Tns3				Gpx4		
Tmed9		Inpp5d		Hsd17b6	Nr2e3				Gsta1		
Spink4		Krt23		Rdh7	Efna4				Gsta4		
Nans		Gprc5c		Alpi	Rnf32				Gstm1		
Cmtm7		Reep5		Gpd1	Prss23				Gstm3		
Creld2		Csk		Ptprh	2010009K17Rik				Gstm6		
Tm9sf3		Bcl2l14		Papss2	Smoc2				H2-Q2		
Wars		Tmem141		Ggt1	Mecom				Hsd17b6		
Smim6		Coprs		Aldh1a1	Esrrg				Ifi27l2b		
Manf		Tmem176b		Naaladl1	Aqp1				Khk		
Oit1		1110007C09Rik		Agpat9	Znrf3				Krt20		
Tram1		Ildr1		H2-Q2	Grb7				Lct		
Kdelr2		Galk1		Hsd17b2	Phgdh				Leap2		
Xbp1		Zfp428		Exoc3l4	2410004N09Rik				Lpgat1		
Serp1		Rgs2		Hpgd	Clca4				Maf		
Vimp		Inpp5b		Gnpda1	Aqp4				Mdh1		
Guk1		Gnai2		Gm1332	Lcp1				Mep1a		
Sh3bgrl3		Pla2g4a		Ms4a10	E030011O05Rik				Mme		
Cmpk1		Acot7		Gm7092	Snhg1				Ms4a10		
Tmsb10		Rbm38		Ugt2a3	BC064078				Mttp		
Dap		Gga2		Upp1	Car12				Muc3		
Ostc		Myo1b		Lrrc19	Zbtb38				Myh14		
Ssr4		Adh1		Fmo4	Cdca7				Myo15b		
Sec61b		Bub3		Hkdc1	Fam13a				Naaladl1		
Pdia3		Sec14l1		Nr1h3	Shisa2				Ndufa1		
Gale		Asah1		Themis3	Dtx4				Neu1		
Klf4		Ppp3ca		Agmo	Slc19a2				Nostrin		
Krtcap2		Agt		Slc6a20a	Fam115c				Nudt4		
Arf4		Gimap1		Soat2	Mir703				Ocm		
15-Sep		Krt18		Ces2a	Cd14				P4hb		
Ssr2		Pim3		Bcl2l15	Mettl20				Phgr1		
Ramp1		2210016L21Rik		Entpd5	Myo9a				Plb1		
Calr		Tmem9		Cndp2	App				Plec		
Ddost		Lima1		Tmem37	Clic6				Pmp22		
		Fam221a		Gda	Wee1				Ppap2a		
