# Supplementary material for: A semi-automatic cell type annotation method for single-cell RNA sequencing dataset
Source: Genomics Inform. 2020 Sep 8;18(3):e26. doi: 10.5808/GI.2020.18.3.e26 (PMC7560448; doi:10.5808/GI.2020.18.3.e26)
Supplement: Supplementary Table 4. — Cell Type Activity score matrix of male and female mouse small intestinal epithelial cell clusters. [file gi-2020-18-3-e26-suppl7.pdf]

Supplementary Table 4. Cell Type Activity score matrix of male and female mouse small intestinal epithelial cell clusters.

	**0**	**1**	**2**	**3**	**4**	**5**	**6**	**7**	**8**	**9**	**10**	**11**	**12**	**13**	**14**
**GOBLET**	0.049048	0.047578	0.050569	0.056945	0.044697	0.044618	0.059116	0.055636	0.216589	0.062009	0.075597	0.061478	0.064323	0.060811	0.050986
**PANETH**	0.05347	0.021958	0.066197	0.030163	0.016176	0.022372	0.048055	0.018656	0.054894	0.039816	0.030953	0.02093	0.50833	0.033449	0.034581
**TUFT**	0.044685	0.038066	0.042407	0.049593	0.056783	0.034462	0.04612	0.04064	0.051797	0.043338	0.062146	0.072701	0.019527	0.334683	0.063053
**ENTEROENDOCRINE**	0.034684	0.021973	0.031057	0.034462	0.028623	0.014284	0.032645	0.011416	0.038199	0.016142	0.616152	0.034045	0.019416	0.036657	0.030246
**ENTEROCYTE**	0.048639	0.059446	0.0493	0.065512	0.021175	0.215306	0.066848	0.128288	0.048965	0.125957	0.048318	0.030327	0.022215	0.04632	0.023383
**STEM**	0.139878	0.074347	0.122798	0.104624	0.023269	0.046124	0.088614	0.04724	0.05026	0.058179	0.051242	0.089412	0.029117	0.050146	0.024749
**TA**	0.097538	0.083834	0.109522	0.162888	0.030891	0.009972	0.128638	0.017658	0.052038	0.022673	0.079179	0.063513	0.017868	0.096047	0.027741
**ENTEROCYTE.PROGENITOR**	0.047252	0.097133	0.050929	0.231063	0.021517	0.027507	0.170903	0.063909	0.055883	0.060074	0.042109	0.045424	0.019292	0.042196	0.02481
**ENTEROCYTE.IMMATURE**	0.053094	0.074454	0.063712	0.07138	0.011918	0.079037	0.0902	0.128924	0.070405	0.13431	0.057487	0.014306	0.054302	0.07339	0.023082
**ENTEROCYTE.MATURE**	0.043886	0.05401	0.045228	0.059146	0.024191	0.256752	0.05931	0.113342	0.053661	0.110235	0.04752	0.030354	0.028573	0.046508	0.027284
