# Supplementary material for: A semi-automatic cell type annotation method for single-cell RNA sequencing dataset
Source: Genomics Inform. 2020 Sep 8;18(3):e26. doi: 10.5808/GI.2020.18.3.e26 (PMC7560448; doi:10.5808/GI.2020.18.3.e26)
Supplement: Supplementary Table 5. — Soft threshold values for network modularization [file gi-2020-18-3-e26-suppl8.pdf]

Supplementary Table 5. Soft threshold values for network modularization

	SOFT THRESHOLD
**B CELL**	4
**DENDRITIC CELL**	3
**ENDOTHELIAL CELL**	3
**FIBROBLAST 1**	2
**FIBROBLAST 2**	6
**GRANULOCYTE**	3
**MACROPHAGE**	6
**NATURAL KILLER CELL**	4
**PERICYTE**	4
**SCHWANN CELL**	5
**SMOOTH MUSCLE CELL**	4
**T CELL**	3
