# Supplementary material for: A semi-automatic cell type annotation method for single-cell RNA sequencing dataset
Source: Genomics Inform. 2020 Sep 8;18(3):e26. doi: 10.5808/GI.2020.18.3.e26 (PMC7560448; doi:10.5808/GI.2020.18.3.e26)
Supplement: Supplementary Table 6. — Gene Ontology term full text of a dendritic cell exemplary module [file gi-2020-18-3-e26-suppl9.pdf]

Supplementary Table 6. Gene Ontology term full text of a dendritic cell exemplary module.

Enrichment	BonferoniP	termID	termName	termDefinition
5.94E-24	1.07E-19	GO:0035456	response to interferon-beta	Any process that results in a change in state or activity of a cell or an organism (in terms of movement, secretion, enzyme production, gene expression, etc.) as a result of an interferon-beta stimulus. Interferon-beta is a type I interferon.
2.15E-20	3.89E-16	GO:0006952	defense response	Reactions, triggered in response to the presence of a foreign body or the occurrence of an injury, which result in restriction of damage to the organism attacked or prevention/recovery from the infection caused by the attack.
3.99E-20	7.21E-16	GO:0045087	innate immune response	Innate immune responses are defense responses mediated by germline encoded components that directly recognize components of potential pathogens.
1.61E-19	2.91E-15	GO:0035458	cellular response to interferon-beta	Any process that results in a change in state or activity of a cell (in terms of movement, secretion, enzyme production, gene expression, etc.) as a result of an interferon-beta stimulus. Interferon-beta is a type I interferon.
4.47E-19	8.08E-15	GO:0043207	response to external biotic stimulus	Any process that results in a change in state or activity of a cell or an organism (in terms of movement, secretion, enzyme production, gene expression, etc.) as a result of an external biotic stimulus, an external stimulus caused by, or produced by living things.
4.47E-19	8.08E-15	GO:0051707	response to other organism	Any process that results in a change in state or activity of a cell or an organism (in terms of movement, secretion, enzyme production, gene expression, etc.) as a result of a stimulus from another living organism.
1.96E-15	3.55E-11	GO:0098542	defense response to other organism	Reactions triggered in response to the presence of another organism that act to protect the cell or organism from damage caused by that organism.
9.01E-15	1.63E-10	GO:0006955	immune response	Any immune system process that functions in the calibrated response of an organism to a potential internal or invasive threat.
2.10E-14	3.80E-10	GO:0034097	response to cytokine	Any process that results in a change in state or activity of a cell or an organism (in terms of movement, secretion, enzyme production, gene expression, etc.) as a result of a cytokine stimulus.
3.64E-14	6.57E-10	GO:0002376	immune system process	Any process involved in the development or functioning of the immune system, an organismal system for calibrated responses to potential internal or invasive threats.
3.52E-13	6.37E-09	GO:0051607	defense response to virus	Reactions triggered in response to the presence of a virus that act to protect the cell or organism.
6.09E-13	1.10E-08	GO:0071345	cellular response to cytokine stimulus	Any process that results in a change in state or activity of a cell (in terms of movement, secretion, enzyme production, gene expression, etc.) as a result of a cytokine stimulus.
1.21E-12	2.19E-08	GO:0009617	response to bacterium	Any process that results in a change in state or activity of a cell or an organism (in terms of movement, secretion, enzyme production, gene expression, etc.) as a result of a stimulus from a bacterium.
2.20E-11	3.98E-07	GO:0009615	response to virus	Any process that results in a change in state or activity of a cell or an organism (in terms of movement, secretion, enzyme production, gene expression, etc.) as a result of a stimulus from a virus.
3.36E-11	6.06E-07	GO:0034340	response to type I interferon	Any process that results in a change in state or activity of a cell or an organism (in terms of movement, secretion, enzyme production, gene expression, etc.) as a result of a type I interferon stimulus. Type I interferons include the interferon-alpha, beta, delta, episilon, zeta, kappa, tau, and omega gene families.
1.47E-10	2.65E-06	GO:0060337	type I interferon signaling pathway	A series of molecular signals initiated by the binding of a type I interferon to a receptor on the surface of a cell, and ending with regulation of a downstream cellular process, e.g. transcription. Type I interferons include the interferon-alpha, beta, delta, episilon, zeta, kappa, tau, and omega gene families.
1.47E-10	2.65E-06	GO:0071357	cellular response to type I interferon	Any process that results in a change in state or activity of a cell (in terms of movement, secretion, enzyme production, gene expression, etc.) as a result of a type I interferon stimulus. Type I interferons include the interferon-alpha, beta, delta, episilon, zeta, kappa, tau, and omega gene families.
5.29E-10	9.56E-06	GO:0045088	regulation of innate immune response	Any process that modulates the frequency, rate or extent of the innate immune response, the organism's first line of defense against infection.
1.25E-09	2.25E-05	GO:0002682	regulation of immune system process	Any process that modulates the frequency, rate, or extent of an immune system process.
1.47E-09	2.66E-05	GO:0035455	response to interferon-alpha	Any process that results in a change in state or activity of a cell or an organism (in terms of movement, secretion, enzyme production, gene expression, etc.) as a result of an interferon-alpha stimulus. Interferon-alpha is a type I interferon.
2.82E-08	0.000509	GO:0045089	positive regulation of innate immune response	Any process that activates or increases the frequency, rate or extent of the innate immune response, the organism's first line of defense against infection.
1.87E-07	0.00338	GO:0019221	cytokine-mediated signaling pathway	A series of molecular signals initiated by the binding of a cytokine to a receptor on the surface of a cell, and ending with regulation of a downstream cellular process, e.g. transcription.
2.25E-07	0.004067	GO:0010033	response to organic substance	Any process that results in a change in state or activity of a cell or an organism (in terms of movement, secretion, enzyme production, gene expression, etc.) as a result of an organic substance stimulus.
2.69E-07	0.004861	GO:0034341	response to interferon-gamma	Any process that results in a change in state or activity of a cell or an organism (in terms of movement, secretion, enzyme production, gene expression, etc.) as a result of an interferon-gamma stimulus. Interferon-gamma is also known as type II interferon.
3.63E-07	0.006562	GO:0002684	positive regulation of immune system process	Any process that activates or increases the frequency, rate, or extent of an immune system process.
5.33E-07	0.009637	GO:0035457	cellular response to interferon-alpha	Any process that results in a change in state or activity of a cell (in terms of movement, secretion, enzyme production, gene expression, etc.) as a result of an interferon-alpha stimulus. Interferon-alpha is a type I interferon.
7.74E-07	0.013989	GO:0071310	cellular response to organic substance	Any process that results in a change in state or activity of a cell (in terms of movement, secretion, enzyme production, gene expression, etc.) as a result of an organic substance stimulus.
1.40E-06	0.025287	GO:0060760	positive regulation of response to cytokine stimulus	Any process that increases the rate, frequency, or extent of a response to cytokine stimulus.
